# Change of neck circumference in relation to visceral fat area: a Chinese community-based longitudinal cohort study

**DOI:** 10.1038/s41366-022-01160-w

**Published:** 2022-06-07

**Authors:** Weijie Cao, Yiting Xu, Yun Shen, Tingting Hu, Yunfeng Xiao, Yufei Wang, Xiaojing Ma, Yuqian Bao

**Affiliations:** 1 Department of Endocrinology and Metabolism, Shanghai Jiaotong University School of Medicine Affiliated Sixth People’s Hospital, Shanghai Clinical Center for Diabetes, Shanghai Key Clinical Center for Metabolic Disease, Shanghai Diabetes Institute, Shanghai Key Laboratory of Diabetes Mellitus, Shanghai, 200233 China; 2 Department of Radiology, Shanghai Jiaotong University School of Medicine Affiliated Sixth People’s Hospital, Shanghai, 200233 China

**Keywords:** Obesity, Nutrition

## Abstract

**Background/Objectives:**

Neck circumference (NC) has been positively associated with visceral fat area (VFA) in cross-sectional studies. This study aimed to evaluate the effects of NC changes on VFA in a Chinese community-based longitudinal cohort.

**Subjects/Methods:**

Subjects recruited from Shanghai communities were followed up for 1.1–2.9 years. A total of 1421 subjects (men 578, women 843) were included, aged 24–80 years, with an average age of 57.8 ± 7.1 years.

**Interventions/Methods:**

Biochemical and anthropometric measurements, including NC, were obtained from all subjects. VFA was assessed by magnetic resonance imaging. Abdominal obesity was defined as a VFA ≥ 80 cm^2^.

**Results:**

After a mean follow-up of 2.1 years, the NCs for men and women were 38.1 ± 2.3 cm and 33.8 ± 2.0 cm, respectively, and the average value of VFA was 84.55 (59.83–113.50) cm^2^. After adjusting for age, sex, body mass index, smoking, history of drinking, glycated hemoglobin, blood pressure and blood lipids, individuals who had gained a NC of more than 5% had 1.26 (95% CI: 1.05–1.49) times more visceral adipose tissue at follow-up than NC maintainers (NC change between –2.5% and 2.5%). In the non-abdominal obesity group at baseline (*n* = 683), after adjusting for confounding factors, changes in NC were associated with abdominal obesity (odd ratio 1.23, 95% CI: 1.09–1.39).

**Conclusions:**

Changes in NC were positively associated with VFA in a Chinese community-based cohort, suggesting that NC measurement is practical for assessing abdominal obesity.

## Introduction

Obesity has been a global public health concern in recent years [1]. Body mass index (BMI) is a simple and convenient index for evaluating the prevalence of obesity [2]. However, obesity defined by BMI has significant heterogeneity, and individuals with similar BMIs may have significantly different comorbidities and health risks. Current studies have shown that the distribution of visceral and ectopic fat plays an important role in cardiovascular metabolic diseases [3, 4]. Computed tomography (CT) and magnetic resonance imaging (MRI) are considered the gold standards for assessing visceral fat area (VFA) [5, 6]. However, due to radiation exposure, time requirements, and high costs, CT or MRI are unsuitable for clinical routines, and a method to evaluate fat distribution quickly and conveniently is still challenging.

As a simple anthropometric index for assessing upper body fat accumulation, neck circumference (NC) measurement is simple and minimally affected by breathing and diet, with an explicit anatomical landmark, high repeatability, and low variability [7]. Our previous cross-sectional study indicated that the optimal cut-off points for NC to estimate abdominal obesity evaluated by MRI were 38.5 cm for men and 34.5 cm for women in Chinese communities [8]. A prospective cohort study including 63 postmenopausal women in Japan found that the change in NC was significantly positively correlated with waist circumference (WC) and body fat after an average follow-up of 3 years [9]. Previous longitudinal studies have indicated that an elevated NC is significantly associated with cardiovascular risk factors such as type 2 diabetes, hypertension, and dyslipidaemia [10–12]. However, a cohort study of the changes in NC and VFA levels has not been reported. The purpose of this study was to explore whether changes in NC were related to VFA and abdominal obesity in a Chinese community cohort in order to provide evidence for the clinical application of NC to assess VFA and abdominal obesity.

## Materials and methods

### Study population

Subjects were enrolled from communities in Shanghai between 2013 and 2014. The collected data were derived from standardized questionnaires and included information on current and previous illnesses, medications, physical examinations, and biochemical measurements. We then conducted a follow-up of the subjects from 2015 to 2016. The subjects were followed up for 1.1–2.9 years with an average of 2.1 ± 0.2 years from 2015 to 2016. We excluded subjects with malignant tumors, steroid hormone or thyroid hormone treatment, abnormal thyroid function or previous hyperthyroidism or hypothyroidism, and history of cardiovascular and cerebrovascular diseases at baseline. A total of 1943 subjects had complete baseline data [8]. Finally, after excluding the losses to follow-up and the lack of follow-up VFAs, a total of 1421 subjects were included in this study.

This study was approved by the Ethics Committee of the Sixth People’s Hospital Affiliated to Shanghai Jiao Tong university of medicine. All subjects provided written informed consent before participation.

### Biochemical measurements

Biochemical variables such as fasting blood glucose (FPG), fasting insulin (FINS), total cholesterol, triglycerides (TG), high-density lipoprotein cholesterol (HDL-C), low-density lipoprotein cholesterol (LDL-C), C-reactive protein, alanine aminotransferase, alanine aminotransferase, alkaline phosphatase, glutamyl transferase, creatinine, and glycated hemoglobin (HbA_1c_) levels were determined from fasting blood samples after an overnight fast using standard methods [8]. Thereafter, subjects without diabetes underwent a 75-g oral glucose tolerance test, and those with diabetes took the 100-g steamed bread meal test; the 2-h blood glucose level was subsequently measured. The homeostasis model assessment of insulin resistance (HOMA-IR) was as follows: HOMA-IR = FINS (mU/l) × FPG (mmol/l)/22.5 [13].

### Anthropometric measurement and visceral fat area

All anthropometric indices and biochemical measurements were collected at baseline and during follow-up. Height, weight, WC, and resting blood pressure were measured using standardized methods [8]. BMI was calculated as weight (kg)/height^2^ (m^2^). NC was measured with the subject standing and the head in the horizontal plane position. The measuring tape was positioned around the inferior margin of the laryngeal prominence and perpendicular to the long axis of the neck. The change in NC (%) was calculated as (NC at follow-up − NC at baseline)/NC at baseline × 100%.

The determination of VFA has been explained in previous studies [8], briefly described as follows: visceral and subcutaneous adipose tissue areas were assessed using a 3.0T clinical MRI scanner (Archiva; Philips Medical System, Amsterdam, The Netherlands), which imaged the abdominal region between the L4 and L5 vertebrae with the subject in the supine position. Segmentation of the images in the VFA and subcutaneous fat area (SFA) was carried out by the Slice-O-Matic image analysis software version 4.2 (Tomovision Inc., Montreal, QC, Canada). Abdominal obesity was defined as a VFA ≥ 80 cm^2^ [14].

### Statistical analyses

Statistical analyses were performed using SPSS 20.0 (IBM Corp., Armonk, NY, USA), and a two-tailed *p* < 0.05, was considered statistically significant. All variables were tested for normality. Normally distributed variables are represented as mean ± standard deviation, and skewed distributed variables are represented by median and interquartile range. A matched samples *t*-test was applied to compare the baseline and follow-up of skewed distributed variables. The Wilcoxon rank-sum test was used to compare skewed distributed variables between the baseline and follow-up. We performed linear regression analyses to examine the association of NC changes with VFA and SFA. Regression coefficients and corresponding 95% confidence intervals (CI) were back-transformed and expressed as ratios, which can be interpreted as relative changes in the measure for abdominal adiposity, compared with that measure in the reference category for NC change. A logistic regression analysis was conducted to analyze the relationship between baseline NC changes and abdominal obesity.

## Results

### Clinical characteristics of study subjects

A total of 1421 subjects (men, 578; women, 843) were aged 24–80 years, with an average of 57.8 ± 7.1 years. The clinical characteristics of the subjects at baseline and follow-up are shown in Table 1. NCs in men and women were 38.2 ± 2.8 cm and 33.7 ± 2.4 cm, respectively. And the average value of VFA was 82.43 (58.27.83–113.10) cm^2^ at baseline. After a mean follow-up of 2.1 years, NCs in men and women were 38.1 ± 2.3 cm and 33.8 ± 2.0 cm, respectively, and the average value of VFA was 84.55 (59.83–113.50) cm^2^. Subjects had significantly higher VFA, FPG, 2-h blood glucose, HbA_1c_, FINS, HOMA-IR, total cholesterol, and HDL-C at follow-up (all *p* < 0.05). There were no significant differences in BMI, NC, WC, SFA, SBP, DBP, TG, LDL-C, and C-reactive protein between baseline and follow-up (all *p* > 0.05) (Table 1) .

**Table 1:** Clinical characteristics of subjects at baseline and follow-up.

Characteristics	Baseline	Follow up
Age (years)	57.8 ± 7.1	59.9 ± 7.1
BMI (kg/m^2^)	24.2 ± 3.2	24.4 ± 3.3
NC (cm) Men	38.1 ± 2.3	38.2 ± 2.8
Women	33.8 ± 2.0	33.7 ± 2.4
WC (cm) Men	87.8 ± 8.9	88.1 ± 8.8
Women	81.9 ± 8.8	82.0 ± 8.9
VFA (cm^2^)	82.43 (58.27–113.10)	84.55 (59.83–113.50)**
SFA (cm^2^)	175.33 (133.71–219.72)	174.75 (133.40–221.65)
SBP (mmHg)	133 ± 18	133 ± 18
DBP (mmHg)	80 ± 11	79 ± 10
FPG (mmol/l)	5.3 (4.9–5.8)	5.8 (5.4–6.5)**
2hPG (mmol/l)	7.3 (5.9–9.4)	7.6 (6.1–9.9)**
HbA_1c_ (%)	5.6 (5.4–5.9)	5.8 (5.5–6.1)**
FINS (uU/ml)	8.1 (5.8–11.7)	9.0 (6.5–13.2)**
HOMA-IR	2.0 (1.3–3.0)	2.4 (1.7–3.7)**
TC (mmol/l)	5.1 ± 0.9	5.4 ± 0.9**
TG (mmol/l)	1.4 (0.9–1.9)	1.5 (1.0–2.1)
HDL-C (mmol/l)	1.3 (1.1–1.6)	1.5 (1.0–2.1)**
LDL-C (mmol/l)	3.2 ± 0.8	3.3 ± 0.9
CRP (mg/l)	0.89 (0.46–1.74)	0.84 (0.46–1.62)
Abdominal obesity, n(%)	785 (55.2%)	848 (59.6%)

Continuous variables are expressed as means ± standard deviation or medians with interquartile range. Categorical variables are expressed as numbers with percentages.

*BMI* body mass index, *SFA* subcutaneous fat area, *VFA* visceral fat area, *NC* neck circumference, *WC* waist circumference, *SBP* systolic blood pressure, *DBP* diastolic blood pressure, *FPG* fasting plasma glucose, *2hPG* 2-h plasma glucose, *FINS* fasting insulin, *HbA*_*1c*_ glycated hemoglobin A_1c_, *HOMA-IR* homeostasis model assessment-insulin resistance index, *TC* total cholesterol, *TG* triglyceride, *HDL-C* high-density lipoprotein cholesterol, *LDL-C* low-density lipoprotein cholesterol.

**p < 0.05.

### Change of NC and VFA and SFA at follow-up

**Fig. 1:**
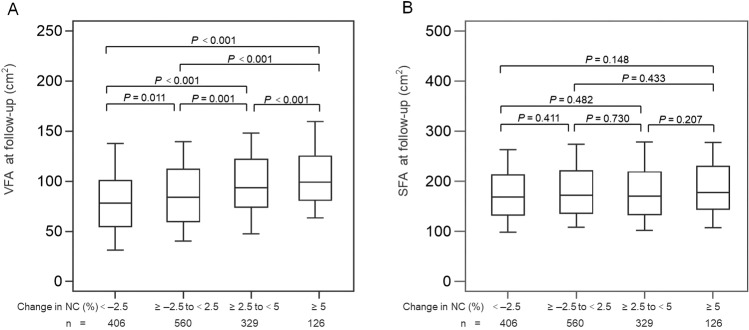
VFA and SFA at follow-up. VFA (**A**) and SFA (**B**) according NC change (%) group (NC change groups are categorized as follows: < –2.5%, ≥ –2.5% to < 2.5%, ≥ 2.5% to < 5% and ≥ 5%) at follow-up. Results are presented as median and interquartile range. The median VFAs (**A**) showed a significant difference in each two groups (all *P* < 0.05) while the median SFAs (**B**) showed no difference between the groups (all P > 0.05).

We categorized NC change as follows: < –2.5%, ≥ –2.5% to <2.5%, ≥2.5% to <5% and ≥5%. The number of subjects in each group were 406 (28.6%), 560 (39.4%), 329 (23.1%), and 126 (8.9%), respectively. The median VFAs of each group were 80.58, 88.5, 97.80 and 101.99 cm^2^ at follow-up, respectively, showing a significant difference in each two groups (all *p* < 0.05), The median SFAs were 168.69, 172.02, 170.66 and 177.34 cm^2^, respectively, showing no difference between the groups (all *p* > 0.05) (Fig. 1).

Linear regression was performed to assess the relationship between NC change and VFA and SFA. NC maintainers (≥ –2.5% to < 2.5%) were used as the reference group. In model 1, after adjusting for sex and age, subjects who had an increased NC of more than 5% showed 1.63 (95% CI: 1.24–1.82) times more visceral adipose tissue than NC maintainers. In model 2, after further adjusting for SBP, DBP, HbA_1c_, HOMA-IR, TG, HDL-C and LDL-C at baseline, subjects who had an increased NC of more than 5% showed 1.42 (95% CI: 1.22–1.60) times more visceral adipose tissue than NC maintainers. In model 3, after further adjusting for baseline BMI and WC, subjects who had and increased NC of more than 5% showed 1.26 (95% CI: 1.05–1.49) times more visceral adipose tissue than NC maintainers. After adjusting for all confounding variables, there was no significant difference of SFA in each NC change group (all *p* > 0.05) (Table 2). Moreover, we stratified subjects into group aged ≥65 years and group aged <65 years to explore influence of age on fat distribution and accumulation. The results were consistent with those of all subjects (Supplementary Table 1).

**Table 2:** Ratios with 95% confidence intervals in measures of VFA and SFA at follow-up by categories of neck circumference change compared with neck circumference maintenance.

	Characteristics neck circumference change categories						
	<–2.5%		≥ –2.5% to <2.5%	≥2.5% to <5%		≥5%	
	Ratio	95% CI	Reference	Ratio	95% CI	Ratio	95% CI
VFA at follow-up							
Model 1	0.91	0.86–0.97	1	1.28	1.08–1.54	1.63	1.24–1.82
Model 2	0.94	0.90–0.99	1	1.26	1.12–1.45	1.42	1.22–1.60
Model 3	0.97	0.94–1.03	1	1.10	1.03–1.25	1.26	1.05–1.49
SFA at follow-up							
Model 1	0.96	0.91–1.02	1	1.14	0.95–1.19	1.14	1.02–1.29
Model 2	0.95	0.91–1.01	1	1.05	0.98–1.09	1.08	1.00–1.17
Model 3	1.02	0.98–1.06	1	1.03	0.99–1.07	1.07	0.96–1.12

Model 1 was adjusted for age and sex. Model 2 was adjusted for model 1 + SBP, DBP, HbA_1c_, HOMA-IR, TG, HDL-C and LDL-C at baseline. Model 3 was adjusted for model 2 + BMI and WC at baseline.

### Change of NC and abdominal obesity

**Fig. 2:**
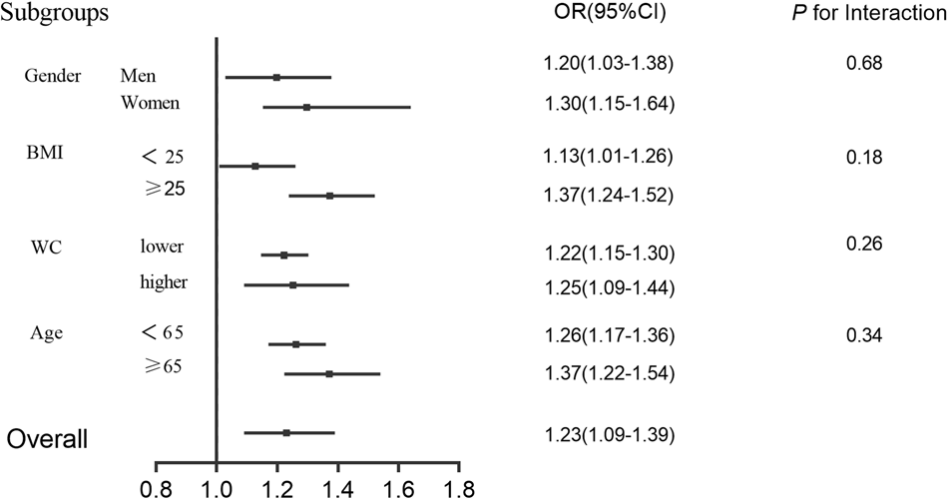
Risk ratio for abdominal obesity. Subgroup analyses by sex (men vs. women), age (<65 vs. ≥65 years), body mass index (BMI < 25 vs. ≥25 kg/m^2^) and waist circumference (WC < 90 cm in men and 85 cm in women vs. WC ≥ 90 cm in men and 85 cm in women using multivariable logistic regression. The model was adjusted for age, sex, BMI, WC, blood pressure, lipid profiles and HbA_1c_. The data are shown as the adjusted odd ratio (95% confidence interval).

Abdominal obesity was defined as a VFA ≥ 80 cm^2^. Logistic regression was used to explore the relationship between changes in NC and abdominal obesity in non-abdominal obesity subjects at baseline (*n* = 683). Using follow-up abdominal obesity as a dependent and independent variable, after adjusting for age, sex, BMI, WC, SBP, DBP, HbA_1c_, TG, HDL-C and LDL-C at baseline, the change of NC (%) was significantly positively correlated with follow-up abdominal obesity (OR: 1.23, 95% CI: 1.09–1.39). As NC increased by 5%, the risk of abdominal obesity increased by 36% (OR: 1.36, 95% CI: 1.19–1.68). After stratification by sex, BMI, WC, and age, the change in NC (%) was significantly positively correlated with follow-up abdominal obesity in each group (all *p* > 0.05) (Fig. 2).

## Discussion

This cohort study indicated that subjects with an increased NC of more than 5% had 1.26 times more visceral adipose tissue than NC maintainers. In subjects with non-abdominal obesity, changes in NC (%) were also closely related to abdominal obesity. With a 5% increase in NC, the risk of abdominal obesity increased by 36%.

Concerning the global obesity pandemic, abdominal obesity or excessive intra-abdominal fat is regarded as the main influencing factor of metabolic abnormalities [14–17]. Numerous cohort studies have shown that VFA, rather than SFA, is closely related to diabetes and cardiovascular events [18, 19]. The increase in VFA is significantly positively correlated with an increased risk of hypertension and dyslipidaemia [20, 21]. Due to racial disparity, there are differences in the VFA cut-offs for assessing abdominal obesity among different races [14, 22]. Our previous cross-sectional study determined VFA by MRI in 1104 community residents and found that VFA ≥ 80 cm^2^ in the Chinese population was the cut-off point for metabolic syndrome and abdominal obesity [14]. The measurement of body fat distribution by MRI or CT is limited by ionizing radiation, time requirements, and high cost. It is not suitable for routine clinical practice in the general population. Therefore, it is urgent to detect a convenient and reliable simple index to evaluate abdominal obesity.

In recent years, with the deepening understanding of anthropometric indicators, NC has been considered to reflect the accumulation of upper body fat [23]. Recently, a Spanish study including 139 adults, aged 18–25 years, accurately evaluated neck fat and abdominal fat tissues using positron emission tomography/CT [24]. It was found that neck fat, such as abdominal fat, was not only significantly correlated with cardiovascular risk factors (blood pressure, blood glucose, blood lipid), but also with cytokines such as interleukin-7, leptin, and adiponectin, suggesting that neck fat and abdominal fat may have similar effects [24]. A prospective cohort study of 63 postmenopausal women in Japan determined that changes in NC were closely related to changes in WC and body fat. Moreover, change in NC was also significantly positively related to the change in pulse wave conduction velocity [9]. Although NC has not been widely used in clinical practice for metabolic diseases, an increasing number of cohort studies have shown that NC can predict the occurrence and development of metabolic diseases, such as metabolic syndrome, diabetes, cardiovascular disease, and fatty liver [10–12, 25].

Various cross-sectional studies have explored the relationship between NC and abdominal obesity. This cross-sectional study assessed VFA by MRI in 1943 community residents and found that there was an independent positive correlation between NC and VFA, demonstrating that NC was an effective simple anthropometric indicator reflecting the degree of abdominal obesity and metabolic abnormalities [8]. Another study of 3182 patients with type 2 diabetes in China found that NC was positively correlated with WC and BMI. Similarly, NC was also significantly associated with abdominal obesity, as defined by WC [10]. In addition, a cross-sectional study of 2234 community residents from South Korea and another with 499 South Africans and Caucasians also confirmed a significant correlation between NC and abdominal obesity [26, 27]. A Dutch study involved 2399 subjects (mean age 57 years) who were asked to recall their weight at the age of 20 years, demonstrated that subjects who gained more than 50% of their body weight showed a 1.96 times increase of VFA and 2.39 times increase of hepatic TG content than weight maintainers [28]. In this study, we investigated the relationship between NC, a simple anthropometric index, and abdominal obesity in a cohort study. NC maintainers (≥2.5% to <2.5%) were set as the reference group, and we found that subjects who had a NC increase of more than 5% showed 1.26 times more visceral adipose tissue than NC maintainers. In subjects with non-abdominal obesity, changes in NC were also closely related to abdominal obesity.

This study had several limitations. Similar to most epidemiological studies, there may have been selection bias. This was a single-center study, which only included a communities Shanghai, and the fat distribution is different in other regions and races. Therefore, further studies based on various regions and races are needed to verify our results. Additionally, aging or other disease may influence cervical spine vertebrae and thin muscles, thus affecting the measurement of NC. The result will be more convincing if we excluded other factors affecting the measurement of NC by imaging examination.

In conclusion, this cohort study of Chinese communities revealed a significant positive correlation between a change in NC (%) and an increase in VFA, suggesting that NC can be a supplementary index for the evaluation of abdominal obesity in clinical practice.

## Supplementary information

Supplementary Table 1

## Data Availability

The data used to support the findings of this study are available from the corresponding author upon request.

## References

[1] Blüher M (2019). Obesity: global epidemiology and pathogenesis. Nat Rev Endocrinol.

[2] Bray GA, Heisel WE, Afshin A, Jensen MD, Dietz WH, Long M (2018). The science of obesity management: an Endocrine Society scientific statement. Endocr Rev.

[3] Klöting N, Fasshauer M, Dietrich A, Kovacs P, Schön MR, Kern M (2010). Insulin-sensitive obesity. Am J Physiol Endocrinol Metab.

[4] Lallukka S, Yki-Järvinen H (2016). Non-alcoholic fatty liver disease and risk of type 2 diabetes. Best Pract Res Clin Endocrinol Metab.

[5] Garvey WT, Mechanick JI, Brett EM, Garber AJ, Hurley DL, Jastreboff AM (2016). American Association of Clinical Endocrinologists and American College of Endocrinology Comprehensive Clinical Practice guidelines for medical care of patients WITH obesity. Endocr Pract.

[6] Omura-Ohata Y, Son C, Makino H, Koezuka R, Tochiya M, Tamanaha T (2019). Efficacy of visceral fat estimation by dual bioelectrical impedance analysis in detecting cardiovascular risk factors in patients with type 2 diabetes. Cardiovasc Diabetol.

[7] Preis SR, Massaro JM, Hoffmann U, D’Agostino RB, Levy D, Robins SJ (2010). Neck circumference as a novel measure of cardiometabolic risk: the Framingham Heart study. J Clin Endocrinol Metab.

[8] Luo Y, Ma X, Shen Y, Xu Y, Xiong Q, Zhang X (2017). Neck circumference as an effective measure for identifying cardio-metabolic syndrome: a comparison with waist circumference. Endocrine.

[9] Aoi S, Miyake T, Iida T, Ikeda H, Ishizaki F, Chikamura C (2016). Association of changes in neck circumference with cardiometabolic risk in postmenopausal healthy women. J Atheroscler Thromb.

[10] Yang GR, Yuan SY, Fu HJ, Wan G, Zhu LX, Bu XL (2010). Neck circumference positively related with central obesity, overweight, and metabolic syndrome in Chinese subjects with type 2 diabetes: Beijing Community Diabetes Study 4. Diabetes Care.

[11] Pumill CA, Bush CG, Greiner MA, Hall ME, Dunlay SM, Correa A (2019). Neck circumference and cardiovascular outcomes: insights from the Jackson Heart Study. Am Heart J.

[12] Roth GA, Mensah GA, Johnson CO, Addolorato G, Ammirati E, Baddour LM, et al. Global burden of cardiovascular diseases and risk factors, 1990-2019: update from the GBD 2019 study [published correction appears in J Am Coll Cardiol. 2021 Apr 20;77(15):1958-1959]. J Am Coll Cardiol. 2020;76:2982–3021. 10.1016/j.jacc.2020.11.010. 10.1016/j.jacc.2020.11.010 PMC7755038 33309175

[13] Matthews DR, Hosker JP, Rudenski AS, Naylor BA, Treacher DF, Turner RC (1985). Homeostasis model assessment: insulin resistance and beta-cell function from fasting plasma glucose and insulin concentrations in man. Diabetologia..

[14] Bao Y, Lu J, Wang C, Yang M, Li H, Zhang X (2008). Optimal waist circumference cutoffs for abdominal obesity in Chinese. Atherosclerosis.

[15] Hayashi T, Boyko EJ, Leonetti DL, McNeely MJ, Newell-Morris L, Kahn SE (2004). Visceral adiposity is an independent predictor of incident hypertension in Japanese Americans. Ann Intern Med.

[16] Hayashi T, Boyko EJ, Leonetti DL, McNeely MJ, Newell-Morris L, Kahn SE (2003). Visceral adiposity and the prevalence of hypertension in Japanese Americans. Circulation.

[17] Neeland IJ, Ross R, Després JP, Matsuzawa Y, Yamashita S, Shai I (2019). Visceral and ectopic fat, atherosclerosis, and cardiometabolic disease: a position statement. Lancet Diabetes Endocrinol.

[18] Sabag A, Way KL, Keating SE, Sultana RN, O’Connor HT, Baker MK (2017). Exercise and ectopic fat in type 2 diabetes: a systematic review and meta-analysis. Diabetes Metab.

[19] González N, Moreno-Villegas Z, González-Bris A, Egido J, Lorenzo Ó (2017). Regulation of visceral and epicardial adipose tissue for preventing cardiovascular injuries associated to obesity and diabetes. Cardiovasc Diabetol.

[20] Sullivan CA, Kahn SE, Fujimoto WY, Hayashi T, Leonetti DL, Boyko EJ (2015). Change in intra-abdominal fat predicts the risk of hypertension in Japanese Americans. Hypertension.

[21] Boyko EJ, Fujimoto WY, Leonetti DL, Newell-Morris L (2000). Visceral adiposity and risk of type 2 diabetes: a prospective study among Japanese Americans. Diabetes Care.

[22] Kvist H, Chowdhury B, Grangård U, Tylén U, Sjöström L (1988). Total and visceral adipose-tissue volumes derived from measurements with computed tomography in adult men and women: predictive equations. Am J Clin Nutr.

[23] Wan H, Wang Y, Xiang Q, Fang S, Chen Y, Chen C (2020). Associations between abdominal obesity indices and diabetic complications: Chinese visceral adiposity index and neck circumference. Cardiovasc Diabetol.

[24] Arias-Tellez MJ, Acosta FM, Garcia-Rivero Y, Pascual-Gamarra JM, Merchan-Ramirez E, Martinez-Tellez B (2021). Neck adipose tissue accumulation is associated with higher overall and central adiposity, a higher cardiometabolic risk, and a pro-inflammatory profile in young adults. Int J Obes.

[25] Shi J, Wang Z, Zhang W, Niu Y, Lin N, Li X (2021). Neck circumference as an independent predictor for NAFLD among postmenopausal women with normal body mass index. Nutr Metab.

[26] Hoebel S, Malan L, de Ridder JH (2012). Determining cut-off values for neck circumference as a measure of the metabolic syndrome amongst a South African cohort: the SABPA study. Endocrine.

[27] Kim KY, Moon HR, Yun JM (2021). Neck circumference as a predictor of metabolic syndrome in Koreans: a cross-sectional study. Nutrients..

[28] Verkouter I, Noordam R, de Roos A, Lamb HJ, Rosendaal FR, van Heemst D (2019). Adult weight change in relation to visceral fat and liver fat at middle age: the Netherlands epidemiology of obesity study. Int J Obes.

