# Supplementary material for: Change of neck circumference in relation to visceral fat area: a Chinese community-based longitudinal cohort study
Source: Int J Obes (Lond). 2022 Jun 7;46(9):1633–7. doi: 10.1038/s41366-022-01160-w (PMC9395262; doi:10.1038/s41366-022-01160-w)
Supplement: Supplementary file 1 — Supplementary Table 1 [file 41366_2022_1160_MOESM1_ESM.docx]

**Supplementary Table 1 Ratios with 95% confidence intervals in measures of VFA and SFA at follow-up by categories of neck circumference change compared with neck circumference maintenance after stratifying by age**

|  | **Characteristics Neck circumference change categories** | | | | | | | | | |
| --- | --- | --- | --- | --- | --- | --- | --- | --- | --- | --- |
|  | ＜–2.5% | |  | ≥ –2.5% to＜2.5% |  | ≥2.5% to＜5% | |  | ≥ 5% | |
|  | **Ratio** | **95%CI** |  | **Reference** |  | **Ratio** | **95%CI** |  | **Ratio** | **95%CI** |
| **VFA at follow-up** |  |  |  |  |  |  |  |  |  |  |
| **Age ≥ 65** | **0.95** | **0.87-1.14** |  | **1** |  | **1.09** | **1.00-1.25** |  | **1.19** | **1.06-1.35** |
| **Age**＜**65** | **0.98** | **0.86-1.19** |  | **1** |  | **1.22** | **1.10-1.32** |  | **1.32** | **1.08-1.54** |
| **SFA at follow-up** |  |  |  |  |  |  |  |  |  |  |
| **Age ≥ 65** | **1.01** | **0.96-1.18** |  | **1** |  | **1.14** | **0.96-1.29** |  | **1.01** | **0.82-1.17** |
| **Age**＜**65** | **1.00** | **0.88-1.13** |  | **1** |  | **1.12** | **0.94-1.30** |  | **0.97** | **0.88-1.12** |

**Model was adjusted for age, sex, SBP, DBP, HbA1c, HOMA-IR, TG, HDL-C, LDL-C, BMI and WC at baseline.**
